# Construction of oxidative phosphorylation-related prognostic risk score model in uveal melanoma

**DOI:** 10.1186/s12886-024-03441-6

**Published:** 2024-05-02

**Authors:** Zhiyun Zhan, Kun Lin, Tingting Wang

**Affiliations:** 1 Ophthalmology Department, First Affiliated Hospital of Fujian Medical University, No. 20, Chazhong Road, Taijiang District, 350004 Fuzhou, Fujian China; 2 Department of Neurosurgery, Shengli Clinical Medical College of Fujian Medical University, 516 Jinrong South Road, 350001 Fuzhou, China

**Keywords:** Uveal melanoma (UVM), Oxidative phosphorylation (OXPHOS), Prognostic risk score model, LASSO regression

## Abstract

**Background:**

Uveal melanoma (UVM) is a malignant intraocular tumor in adults. Targeting genes related to oxidative phosphorylation (OXPHOS) may play a role in anti-tumor therapy. However, the clinical significance of oxidative phosphorylation in UVM is unclear.

**Method:**

The 134 OXPHOS-related genes were obtained from the KEGG pathway, the TCGA UVM dataset contained 80 samples, served as the training set, while GSE22138 and GSE39717 was used as the validation set. LASSO regression was carried out to identify OXPHOS-related prognostic genes. The coefficients obtained from Cox multivariate regression analysis were used to calculate a risk score, which facilitated the construction of a prognostic model. Kaplan-Meier survival analysis, logrank test and ROC curve using the time “timeROC” package were conducted. The immune cell frequency in low- and high-risk group was analyzed through Cibersort tool. The specific genomic alterations were analyzed by “maftools” R package. The differential expressed genes between low- or high-risk group were analyzed and performed Gene Ontology (GO) and GSEA. Finally, we verified the function of CYC1 in UVM by gene silencing in vitro.

**Results:**

A total of 9 OXPHOS-related prognostic genes were identified, including NDUFB1, NDUFB8, ATP12A, NDUFA3, CYC1, COX6B1, ATP6V1G2, ATP4B and NDUFB4. The UVM prognostic risk model was constructed based on the 9 OXPHOS-related prognostic genes. The prognosis of patients in the high-risk group was poorer than low-risk group. Besides, the ROC curve demonstrated that the area under the curve of the model for predicting the 1 to 5-year survival rate of UVM patients were all more than 0.88. External validation in GSE22138 and GSE39717 dataset revealed that these 9 genes could also be utilized to evaluate and predict the overall survival of patients with UVM. The risk score levels related to immune cell frequency and specific genomic alterations. The DEGs between the low- and high- risk group were enriched in tumor OXPHOS and immune related pathway. In vitro experiments, CYC1 silencing significantly inhibited UVM cell proliferation and invasion, induced cell apoptosis.

**Conclusion:**

In sum, a prognostic risk score model based on oxidative phosphorylation-related genes in UVM was developed to enhance understanding of the disease. This prognostic risk score model may help to find potential therapeutic targets for UVM patients. CYC1 acts as an oncogene role in UVM.

**Supplementary Information:**

The online version contains supplementary material available at 10.1186/s12886-024-03441-6.

## Introduction

Uveal melanoma (UVM) is the predominant malignant intraocular tumor in adults [1, 2]. Its incidence rate ranks first among intraocular tumors abroad and second only to retinoblastoma in China [3–5]. This highly malignant tumor exhibits a propensity for hematogenous metastasis and primarily affects adults [6, 7]. Clinically, it can be challenging to differentiate from various fundus diseases. Despite the high success rate of local UVM treatment, 50% of patients eventually develop tumor metastasis [8]. Therefore, full attention should be paid in the clinical work of uveal melanoma.

In the process of biological oxidation, substrate dehydrogenation produces NADH and FMNH2, which are oxidized through the respiratory chain to generate water [9, 10]. Simultaneously, the free energy released is used to couple ADP phosphorylation to generate ATP [11]. This coupling effect between oxidation and phosphorylation is called oxidative phosphorylation (OXPHOS). Studies have found that the glycolysis level of tumor cells is higher compared with normal cell, suggesting that the oxidative phosphorylation level in tumor cells may be abnormal [12]. For example, oxidative phosphorylation levels are elevated in some tumors, including leukemia [13], lymphoma [14], pancreatic ductal adenocarcinoma [12], and endometrial cancer [15]. Targeting genes related to oxidative phosphorylation may play a role in anti-tumor therapy, and may target tumor cell metabolism to play an anti-tumor role [12].

In recent years, the risk model based on multiple genes has been widely studied and used to predict the prognosis of various tumors, such as colon cancer, breast cancer, hepatocellular carcinoma, etc. In some cancer species, its prognosis prediction performance is even better than histopathological diagnosis and tumor staging [16]. Least absolute shrinkage and selection operator (LASSO) is a data mining method, in which a penalty function is added to the commonly used multiple linear regression to continuously compress the coefficients, so as to achieve the purpose of simplifying the model to avoid collinearity and overfitting [17]. At present, Cox regression based on LASSO method is widely used in tumor prognostic signature screening and risk model construction, which has obvious advantages over traditional methods [18]. However, it is mostly used to screen gene signatures in the study of prognostic factors of UVM, while there are few studies on constructing prognostic models after screening clinical and pathological variables.

In this study, bioinformatics was used to screen relevant genes with prognostic value for UVM based on the genes related to oxidative phosphorylation in KEGG. Through lasso and Cox regression analysis, a prognostic risk scoring model of UVM composed of 9 OXPHOS-genes was finally constructed to assess the predictive ability of the prognostic risk score model. The independent prognostic value and clinical relevance of the model were further determined. This research provides deeper understanding for the individualized diagnosis and treatment of UVM patients.

## Method

### Data collection and processing

The Oxidative phosphorylation related genes were obtained from the KEGG Oxidative phosphorylation pathways, which included 134 genes. The TCGA Ocular Melanomas (UVM) dataset contained 80 samples [19] was downloaded from UCXC Xena. Somatic mutation and copy number alternation (CNA) data were also downloaded from the TCGA database. The GSE22138 was downloaded from GEO database, which contained transcriptome of 63 uveal melanoma from enucleation of untreated patients [20]. GSE39717 dataset was downloaded from Gene Expression Omnibus, included 39 primary UVM tissue samples and 2 metastatic UVM tissue samples. The GSE22138 and GSE39717 datasets were used to enhance the clarity of the relationship between OXPHOS genes and UVM cancer. The samples were normalized using quantile normalization.

### Least absolute shrinkage and selection operator (Lasso) regression

Lasso regression is also known as lasso algorithm. It can adjust the regression coefficient of independent variables, and even compress the variable coefficient that has little impact on the model to zero, thus reducing the overfitting of data to a certain extent, and screening out relatively important variables [21]. Through the “glmnet” package and “survival” package in R language, the OXPHOS-related genes screened by single factor was further analyzed by lasso regression, and the calculated minimum λ value as the best reference value, represents the best variable included in the model, to obtain OXPHOS-related genes that is more relevant to the prognosis of UVM patients.

### Construction and validation of the prognostic risk score model

The Cox multivariate regression analysis coefficient of prognostic gene was extracted, and the risk score was calculated by the following formula using the gene expression level as the score of survival risk of each patient:

Risk score = Coef1 × expr mRNA1 + Coef2 × expr mRNA2+⋯+ Coefn × expr mRNAn.

Coefn represents the Cox risk proportional coefficient of mRNAn, and expr mRNA represents the expression level of the gene. All samples of TCGA were divided into high-risk and low-risk groups according to the risk score, with the median risk score being the threshold.

GSE22138 was used to evaluate the effectiveness and robustness of the above prognostic risk model. In the same way, each group of samples was divided into high-risk and low-risk groups according to the risk score.

Survival differences between different risk groups in each group were analyzed using Kaplan– Meier curve combined with logrank test. Using the time “timeROC” package, the area under the ROC was determined, and 1-year to 5-year survival rates were predicted respectively.

### Identification of differential expressed genes (DEGs)

The DEGs between low- or high-risk group were analyzed by limma R package. Using the gene annotation file in gencode, the gene ID was transformed into gene symbol, and the DEGs were identified. At the same time, volcano map and heat map are drawn to visually show the expression of differential DEGs. The heat map is drawn using the “pheatmap” R package, and the volcano map is drawn using the “ggplot2” R package.

### Functional enrichment analysis

The “clusterprofiler” and “enrichlot” packages of R software were carried out for Gene Ontology (GO) enrichment analysis and visualization of differential genes to explore the potential molecular mechanism of DEGs with prognostic value. Gene set enrichment analysis is based on functional annotation or previous experimental results, which integrates the differential expression sets in two samples. The KEGG datasets and hallmark datasets are selected for analysis. *P* < 0.05 was considered statistically significant.

### Cell culture and transfection

Human uveal melanoma cell lines MUM-2 C was obtained from the Center for Type Culture Collection of China. Cells were cultured in RPMI-1640 medium supplemented with 10% fetal bovine serum (FBS; HyClone, Grand Island, NY, USA) and penicillin/streptomycin (Sangon, Shanghai, China) at 37℃ in a 5% CO_2_ incubator.

For transfection, the plasmid was then used to construct the short hairpin RNA (shRNA) for CYC1 downregulation. Transient transfection was performed with Lipofectamine 2000 Transfection Reagent (Invitrogen, Carlsbad, CA, USA) as protocols.

### Real-time polymerase chain reaction (qPCR)

RNA extraction of MUM-2 C cells was performed using TRIzol Reagent (Invitrogen). 2ug of RNA was reverse-transcribed into cDNA and the M-MLV Reverse Transcriptase was used (Invitrogen). Real-time PCR was conducted on a Real-Time PCR system (Bio-Rad, Hercules, CA, USA). Primers of CYC1 used for amplification were as follows: CYC1 5′-AGCTATCCGTGGTCTCACC-3′ and 5′-CCGCATGAACATCTCCCCATC-3′.

### Clone formation assay

For the colony formation assay, MUM-2 C cells were plated onto 10 cm dishes at a density of 5 × 10^4^ cells per dish. Colonies composed cells were counted 10–14 days after seeding under the microscope (40×). The colonies were then stained for 30 min. 0.5% crystal violet in 20% methanol was used for staining and photographed. All experiments were performed in triplicate wells three times.

### Cell invasion assays

The transwell chambers were solidified with Matrigel (BD Biosciences, San Jose, CA, USA) by incubation at 37℃ for 6 h. cells were seeded into the upper chamber in serum-free RPMI-1640 medium. Meanwhile, medium containing 10% FBS was added in the lower chamber. After 24 h, invasive cells on the lower chamber were stained and counted.

### Flow cytometry analysis

MUM-2 C cells were cultured in 6-well plates after transfection. Followed by rinsing with PBS, Annexin V-FITC and Propidium iodide (PI) were used for double staining. Apoptosis rate was measured via a flow cytometry (BD Biosciences, Beijing, China).

### Statistical analysis

All statistical analyses were performed using SPSS 22.0 or R software. SPSS 22.0 (IBM Corporation, NY, USA) was used for statical analysis. The dates are expressed as means ± standard deviation. One way ANOVA test was used to compare the mean between multiple samples, The Student’s t-test was used to compare the mean between two samples. *P* < 0.05 was considered statical significance.

## Results

### Identification of OXPHOS-related prognostic genes in UVM

The 134 OXPHOS-related genes were obtained from the Oxidative phosphorylation pathway in KEGG sets. We performed univariate Cox regression analysis based on these 134 genes and screened out 42 oxidative phosphorylation (OXPHOS)-genes with prognostic significance (Figure S1, p value < 0.05), in which 38 genes (NDUFB1, NDUFB8, CYC1, etc.) presented poor prognostic role in UVM (Hazar Ratio, HR > 1). It suggests the high expression of these genes is associated with high risk.

### Construction and evaluation of prognostic risk score model

**Fig. 1:**
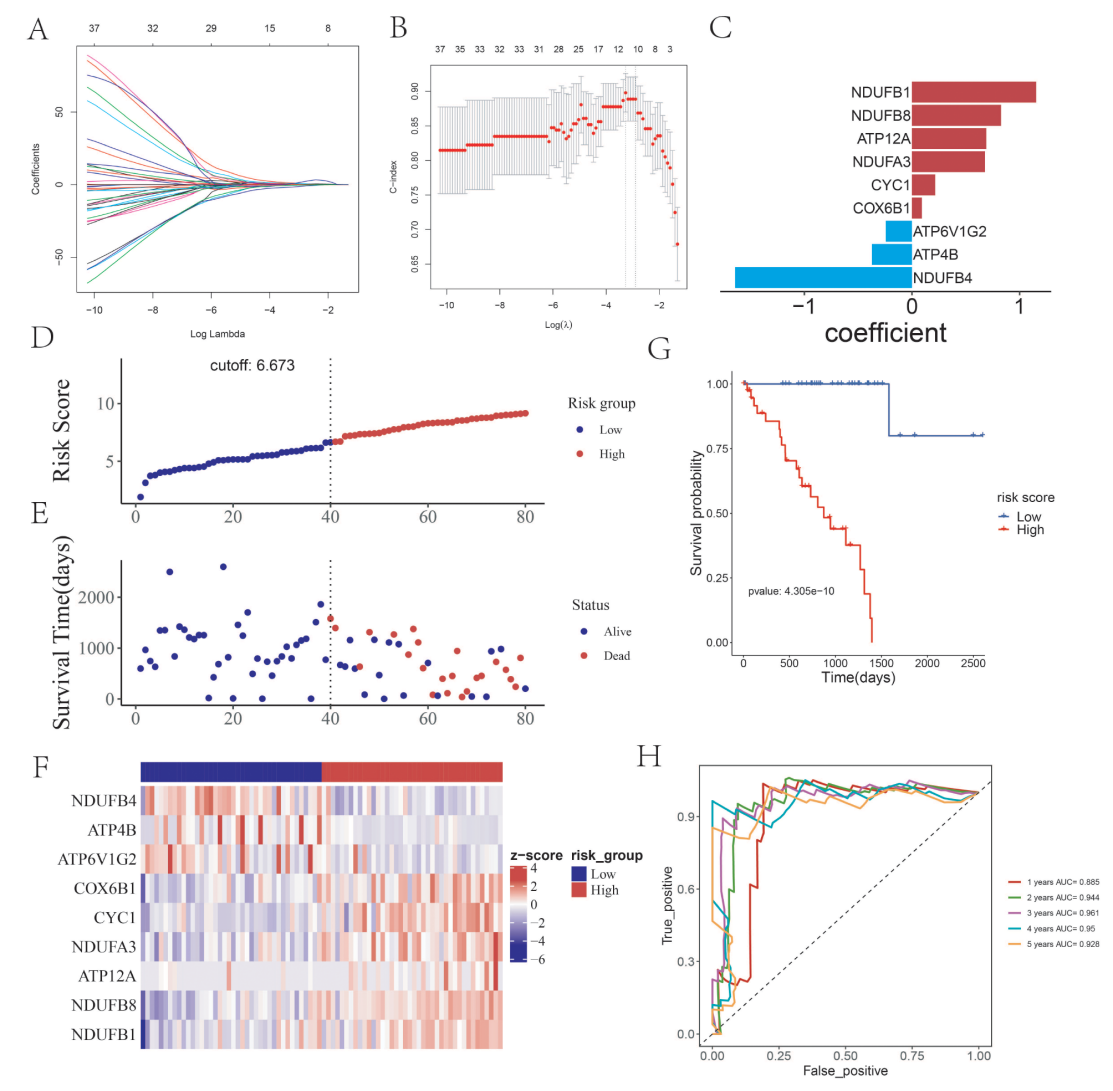
Construction of prognostic risk score model of the Oxidative phosphorylation (OXPHOS)-related genes based on the TCGA database. (**A**-**B**) LASSO regression analysis. (**C**) The coefficient of 9 identified genes, including NDUFB1, NDUFB8, ATP12A, NDUFA3, CYC1, COX6B1, ATP6V1G2, ATP4B and NDUFB4. (**D**) The risk score curve of the samples in high-risk group and low-risk group. The cutoff value is 6.637. (**E**) The survival status chart; of samples in high-risk group and low-risk group. (**F**) The heatmap showing the expression level of the 9 OXPHOS-related signature genes (**G**) The Kaplan-Meier survival curve of high-risk group and low-risk group. (**H**) The one to five-year time ROC curve of risk score in the TCGA training set

The lasso regression analysis could be used to reduce the overfitting of the data and minimize the λ Value as the best reference value to screen genes that are more critical to prognosis. In the next step, the lasso regression analysis was performed based on the above 42 genes through the “glmnet” and “survival” R package (Fig. 1A-B). A total of 9 more stable OXPHOS-related prognostic genes were identified, which including NDUFB1 (Coef = 1.153), NDUFB8 (Coef = 0.8266), ATP12A (Coef = 0.6900), NDUFA3 (Coef = 0.6783), CYC1(Coef = 0.2152), COX6B1(Coef = 0.09137), ATP6V1G2 (Coef=-0.2422), ATP4B (Coef=-0.3718) and NDUFB4 (Coef=-1.6423) (Fig. 1C). Survival analysis was performed for 9 genes in the prognostic risk model constructed in this study in the TCGA UVM database according to the median grouping. The Kaplan-Meier survival analysis of the 9 OXPHOS-related signature genes were shown in Figure S2. NDUFB4, ATP4B and ATP6V1G2 were associated with better prognosis and survival of UVM, while NDUFB1, NDUFB8, ATP12A, NDUFA3, CYC1, COX6B1were associated with poor prognosis and survival of UVM.

The prognostic risk model of UVM was constructed based on the 9 OXPHOS-related prognostic genes. At the same time, the risk value of each sample was calculated, and the median of the risk score was taken as the cutoff value (cutoff value = 6.673), which was divided into high-risk group and low-risk group (Fig. 1D and E). The probability distribution diagram of risk score and the heat map of 9 OXPHOS-related prognostic genes expression in high and low-risk groups were also presented (Fig. 1F).

The prediction ability of the prognostic risk score model was evaluated by Kaplan-Meier survival analysis and ROC curve. The results showed that the prognosis of patients in the high-risk group was poor, and the difference was statistically significant (Fig. 1G, *p* = 4.305e-10). Besides, the ROC curve demonstrated that the area under the curve of the model for predicting the 1 to 5-year survival rate of UVM patients were all more than 0.88 (AUC = 0.885, 0.944, 0.961,0.95 and 0.927, respectively), which revealed the accuracy of the model (Fig. 1H).

### Performance evaluation of prognostic risk score model in GSE22138 and GSE39717 datasets

**Fig. 2:**
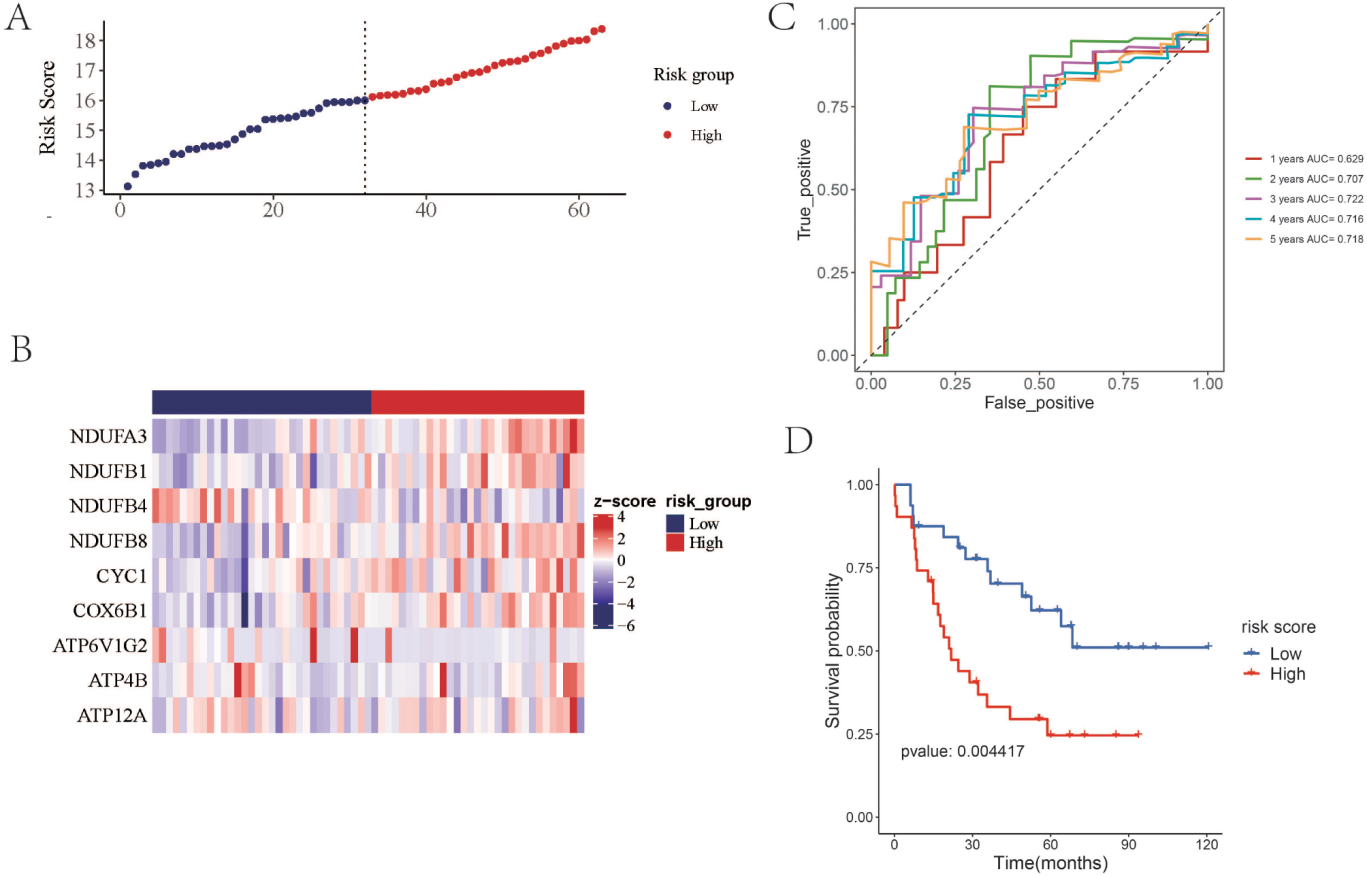
Performance evaluation of prognostic risk score model in external validation sets GSE22138. (**A**) The risk score of the samples in high-risk group and low-risk group. (**B**) The heatmap showing the expression level of the 9 OXPHOS-related signature genes. (**C**) The Kaplan-Meier survival curve of high-risk group and low-risk group. (**D**) The one to five-year Time ROC curve

In the next step, we evaluated the performance of prognostic risk score model in GSE22138 dataset. According to multivariate Cox regression analysis, a risk score was calculated for each patient using the 9 OXPHOS-related prognostic genes, and the cutoff value separating high-risk and low-risk groups was set at the median (16.213) (Fig. 2A). The expression trend of the 9 OXPHOS-related prognostic genes in high and low risk groups was consistent with the TCGA train set (Fig. 2B). The prediction performance of six genes was evaluated by using the time-dependent ROC curve for GSE22138. As seen in Fig. 2C. The higher the AUC, the better the model performance. The AUC of the external data set was slightly lower than that of the TCGA data sets. Kaplan-Meier survival curves showed significant differences between groups, the prognosis of high-risk group was significantly lower than those of the low-risk group (Fig. 2D). External validation revealed that these 9 genes could also be used to evaluate and predict the overall survival of patients with UVM.

Further, we evaluated the performance of prognostic risk score model in GSE39717 dataset. According to multivariate Cox regression analysis, a risk score was calculated for each patient using the 9 OXPHOS-related prognostic genes, and the cutoff value separating high-risk and low risk groups was set at the median (14.598) (Figure S3A). The expression trend of the 9 OXPHOS -related prognostic genes in high and low risk groups was consistent with the TCGA train set (Figure S3A). To explore the predictive value of the signature, the Kaplan-Meier survival curves were analyzed. UVM patients of high-risk group has shorter OS (Figure S3B). The prediction performance of six genes was evaluated by using the time-dependent ROC curve for GSE39717. ROC analysis confirmed that the area under curve (AUC) was 0.653 at 1 years, 0.834 at 2 years, 0.742 at 3 years, 0.772 at 4years, and 0.653 at 5 years, respectively (Figure S3C). AUC values were more than 0.5 regardless of the predicted survival time at 1, 2, 3, 4, 5-year survival in the GSE39717 dataset.

### The risk score levels were associated with immune cell frequency and specific genomic alterations

**Fig. 3:**
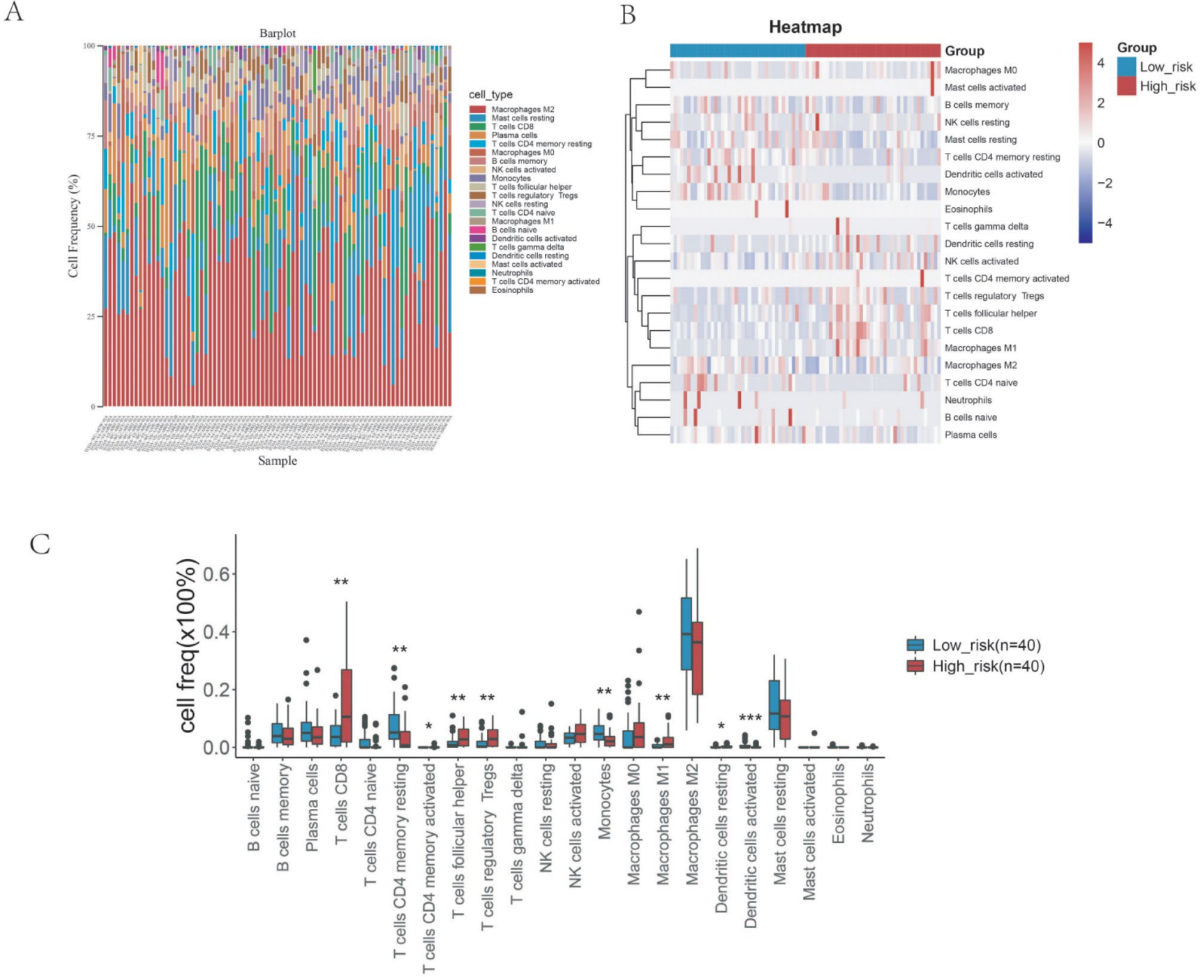
The immune cell frequency in samples and low- and high-risk group. (**A**) The cell frequency of immune cell in all samples. (**B**) The heatmap showing the relative expression level of samples in low- and high-risk in immune cell. (**C**) The cell frequency of immune cell between the low- and high-risk group

We next analyzed the immune cell frequency in samples and low- and high-risk group through Cibersort. As presented in Fig. 3A and B, the frequency of different immune cell types in each sample were presented, in which we found that Macrophages M2, Mast cells resting, T cell CD8, plasma cell and T cells CD4 memory resting were occupied most of the frequency. What’s more, the T cell CD8, T cells CD4 memory resting, T cell follicular helper, T cell regulatory Treg, Monocytes, Macrophages M1 Dendritic cells resting and Dendritic cells active were significantly expressed differently between the high- and low risk group (Fig. 3C).

**Fig. 4:**
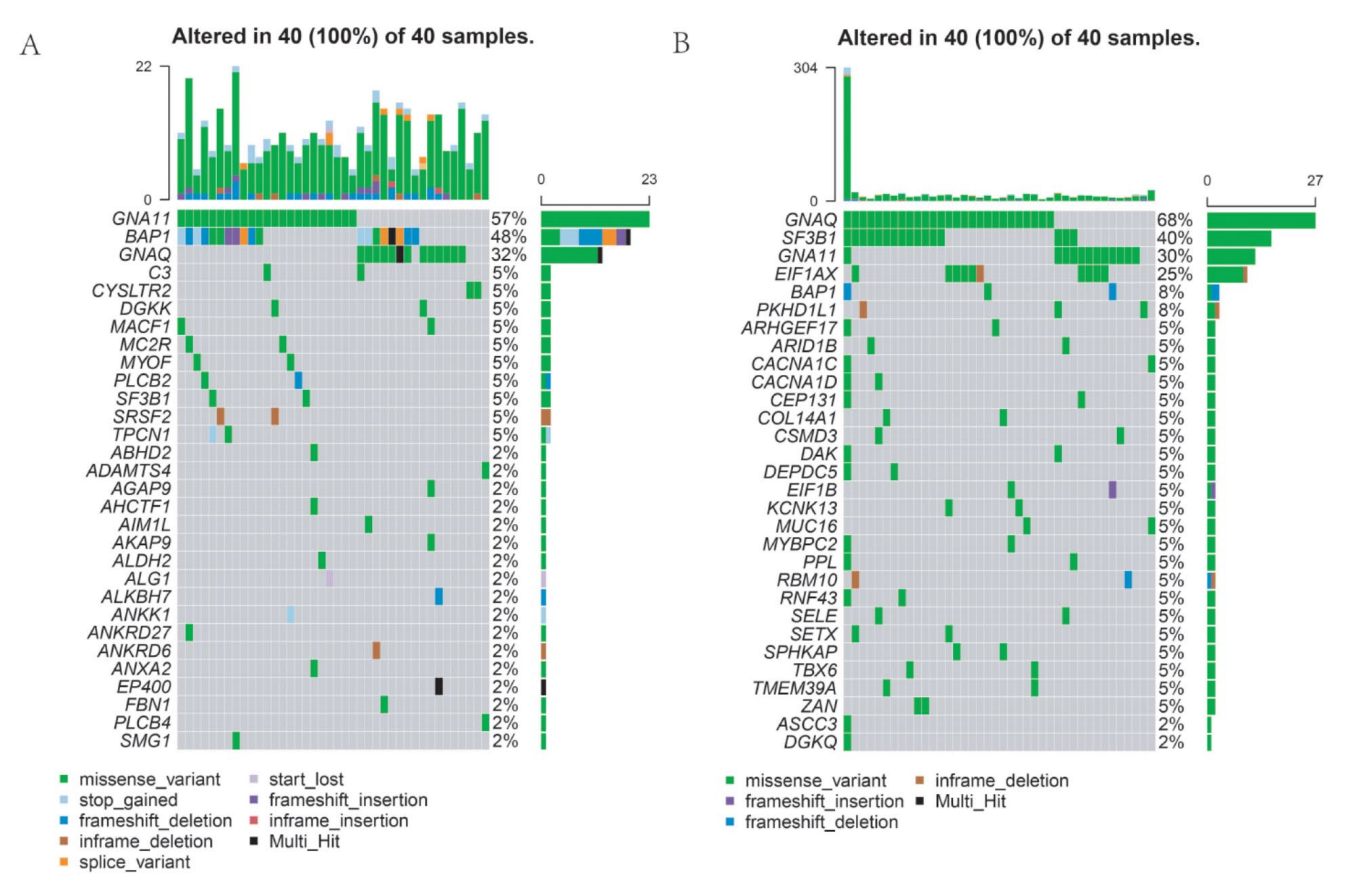
Genomic alterations in score-low versus score-high clusters. (**A**-**B**) Themaftool exhibited incidence of somatic mutations of UVMs in 40 UVM patients from of the high-risk group (**A**) and low-risk group cluster (**B**)

To investigate the association of risk score level and specific genomic mutational signatures, the somatic mutational assays was conducted based on the TCGA information. Next, we conducted general analysis of the somatic mutation frequency in these 40 low/high risk UVMs, the result showed a relatively high mutation frequency in UVM samples (Fig. 4A and B). In detail, 40(100%) UVM samples had mutations in UVM of high/low risk groups. Bioinformatics of somatic mutation profiles based on risk score levels showed the increased frequencies of mutations in GNA11 (57%), BAP1 (48%), and GNAQ (32%) in the high-risk group (*n* = 40), while GNAQ (68%), SF3B1 (40%), GNA11 (30%), and EIF1AX (25%) were more frequently mutated in the low-risk group (*n* = 40) (Fig. 4A and B).

### The DEGs between the low- and high- risk group were enriched in tumor OXPHOS and immune related pathway

**Fig. 5:**
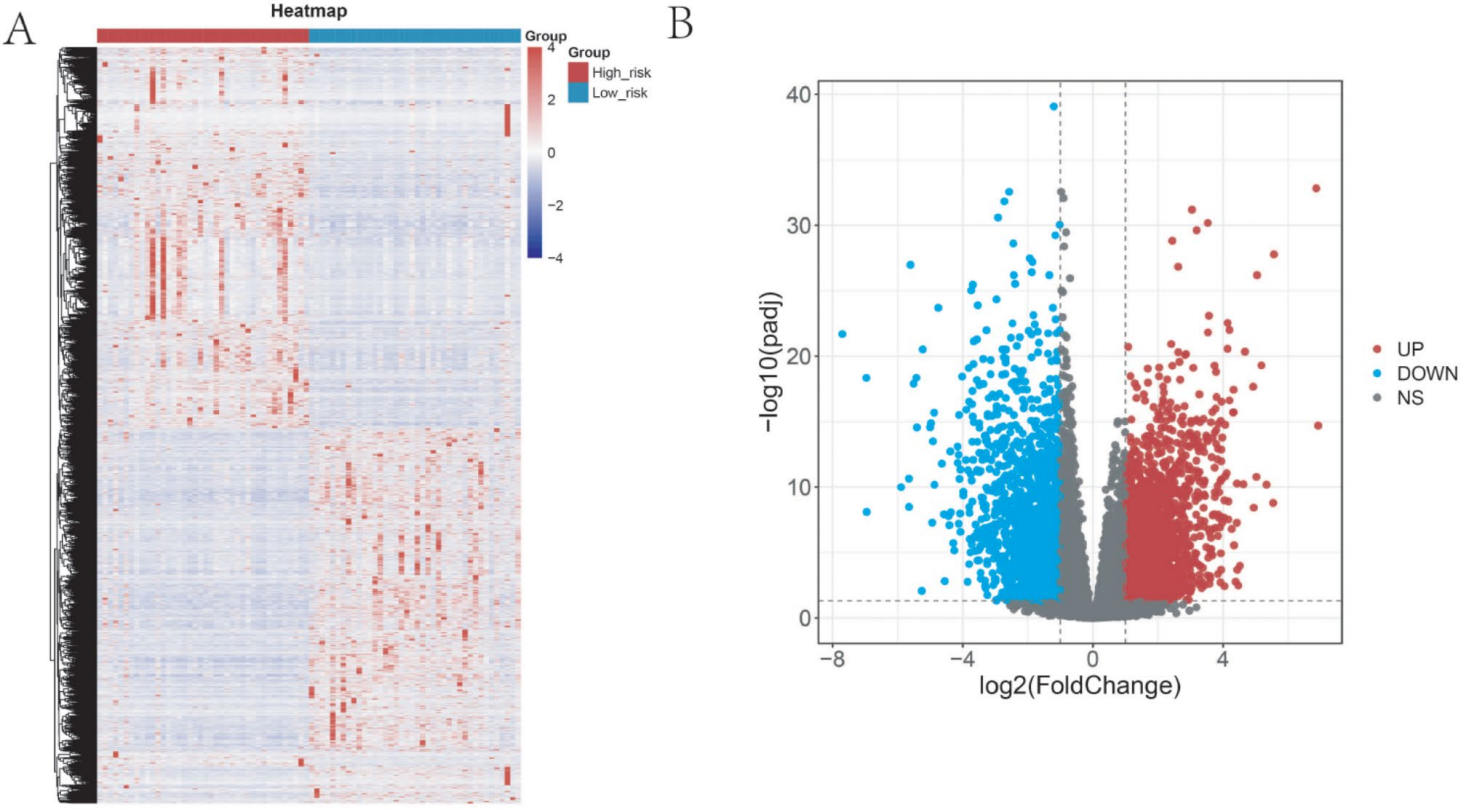
The differential expressed genes (DEGs) between the high-risk group and low-risk group. (**A**) The heatmap showing the expression level of DEGs. The screening criteria is|log2FC| > = 1 and p. adjust < = 0.05. (**B**) The volcano plot presented the DEGs, the red dots indicated up-regulated DEGs and blue dots indicated down-regulated DEGs

**Fig. 6:**
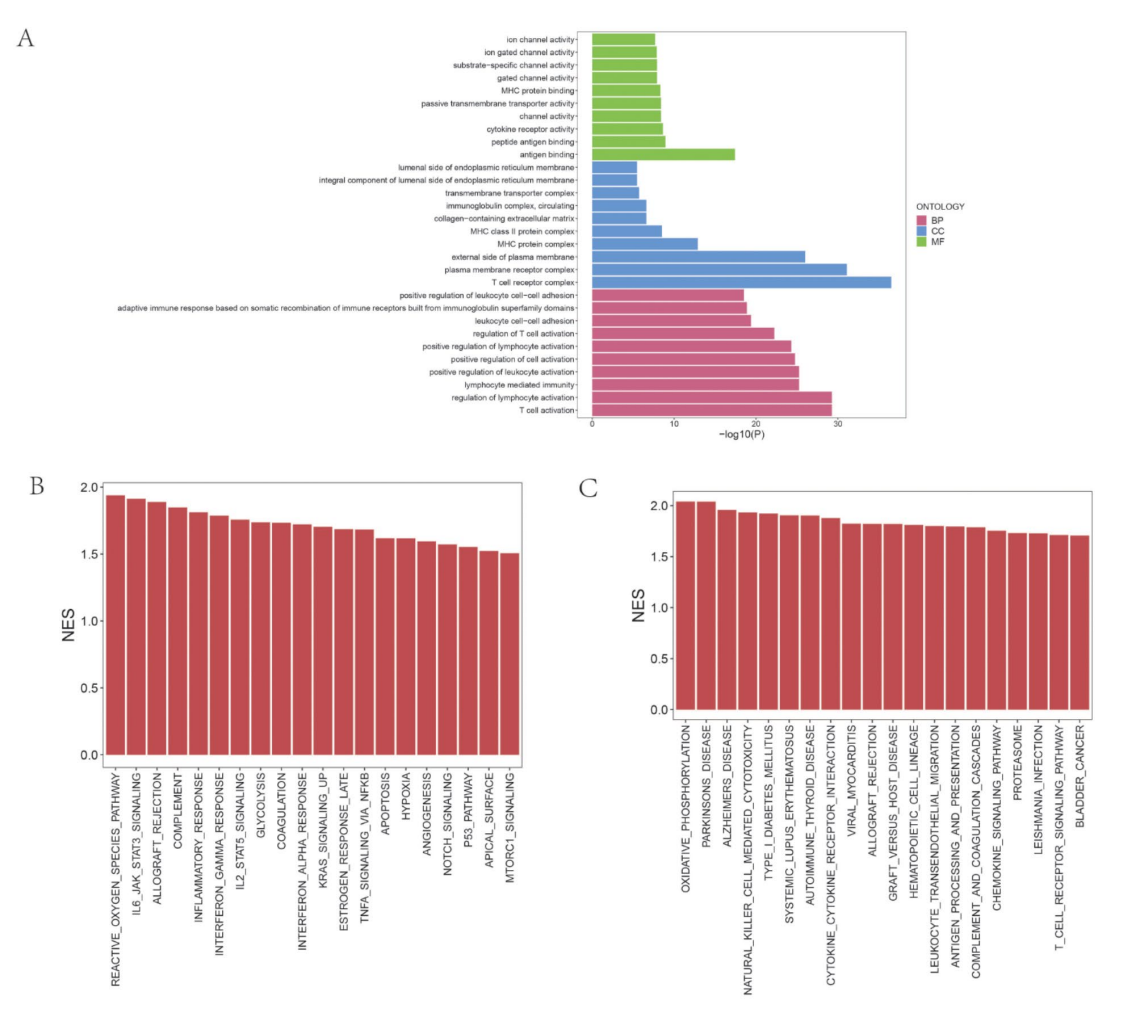
The functional enrichment analysis of DEGs between the low- and high-risk group. (**A**) The Gene Ontology (GO) functional enrichment of DEGs, which included the Biological Process (BP), Cell Component (CC), Molecular Function (MF). (**B**-**C**) The Gene set enrichment analysis (GSEA) of DEGs based on HALLMARK pathways set (**B**) and KEGG set (**C**)

To explore the underlying molecular mechanism, we sequentially identified the DEGs between low- and high- risk group. As seen in Figs. 5A and B and 4566 DEGs were screened out, which included 2400 up-regulated DEGs and 2166 down-regulated DEGs. Thereafter, the GO and GSEA enrichment analysis were carried out. As seen in Fig. 6A, the DEGs were enriched in biology process of cell − cell adhesion, regulation of lymphocyte and T cell activation, cell component of T cell receptor complex. The GESA analysis of HALLMARK presented that the DEGs were enriched in reactive oxygen species pathway, IL6-JAK-STAT3, KRAS, TNFA- NFKB, NOTCH, P53, and MTORC1 signaling (Fig. 6B). Besides, in the GSEA analysis of the KEGG pathway, the DEGs were enriched in oxidative phosphorylation, natural killer cell mediated cytotoxicity, leukocyte transendothelial migration, T cell receptor signaling pathway, etc. (Fig. 6C).

### Knockdown of CYC1 inhibited cell proliferation, invasion and induced cell apoptosis in UVM cells

**Fig. 7:**
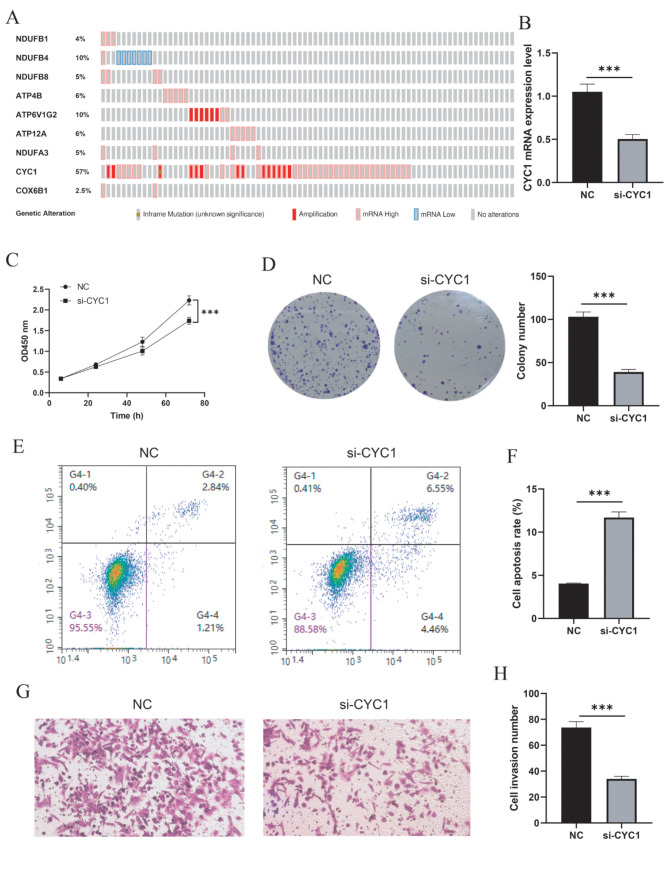
Effects of CYC1 on the proliferation, invasion and apoptosis of MUM-2 C cells in vitro. (**A**) The copy number alteration frequency of OXPHOS related genes in UVM. (**B**) CYC1 expression was analyzed by RT-qPCR assay. (**C**) CCK-8 assay detected the MUM-2 C cells proliferation. (**D**) Colon formation assay was used to detected cell growth number of MUM-2 C cells. (**E**) Glow cytometry assay was used to detect the apoptosis of MUM-2 C cell lines. (**F**) Cell apoptosis rate was calculated. (**G**) Transwell assay was performed to explore the invasion of MUM-2 C cell. (**H**) Cell invasion number was calculated. Data are represented as mean ± SD. * *P* < 0.05, ** *P* < 0.01, ***P < 0.001

cBioPortal shown that the copy number alteration (CNA) of 9 OXPHOS related genes in UVM. The amplification and CAN frequency of CYC1 (57%), ATP6V1G2 (10%), ATP4B(6%), ATP12A(6%), NDUFA3(5%), NDUFB8(5%), NDUFB1 (4%), COX6B1 (2.5%), CYC1 amplification was most significant (Fig. 7A). The role of CYC1 in UVM cells is still unclear. We investigated the role of CYC1 in UVM MUM-2 C cells. First, RT-qPCR assays showed that CYC1 expression was significantly reduced in si-CYC1 group (Fig. 7B). Knockdown of CYC1 inhibited the proliferation of MUM-2 C cell lines, as shown by the CCK-8 assay (Fig. 7C). Colon formation assay results shown that knockdown of CYC1 decreased the cell growth number of MUM-2 C cell lines (Fig. 7D). Next, we analyzed the effect of CYC1 on the apoptosis of MUM-2 C cell lines using flow cytometry. We found that knockdown of CYC1 increased the cell apoptotic rates (Fig. 7E and F). In addition, transwell assay revealed that cell invasion was reduced in the si-CYC1 group compared with the control group (Fig. 7G and H). Collectively, these results suggested that knockdown of CYC1 inhibited cell proliferation, invasion and promoted cell apoptosis in UVM cells.

## Discussion

One of the current challenges in the treatment of UVM is that the prognosis of patients who progress to metastatic disease is still poor [22]. Some researchers have provided formulas for the combination of clinical and genetic parameters. For example, Wang et al. used WGCNA for co-expression modules and identify potential prognostic markers of UVM [23]. Cao et al. screened out for 10 genes to constructed prognostic Implications in UVM [24]. Ma et al. identified the ferroptosis-related gene to assess prognosis in UVM [25].

Oxidative phosphorylation (OXPHOS) provides most of the ATP used by advanced animals and plants to maintain life and is responsible for maintaining metabolic homeostasis [26]. The bioenergy of some tumors is dependent on OXPHOS [27, 28]. What’s more, biomass production is important for enhancing tumor development, which indicates that targeted oxidative phosphorylation will be an effective cancer treatment [29]. However, the underlying mechanism of oxidative phosphorylation in UVM has not been explored yet.

In this research, we identified 9 OXPHOS-related prognostic genes to constructed the risk score model of UVM, including NDUFB1, NDUFB4, NDUFB8, ATP4B, ATP6V1G2, ATP12A, NDUFA3, CYC1, and COX6B1. These OXPHOS-related genes are related with mitochondrial metabolism [30]. Among the night genes, CYC1 has been previously studied in UVM, and the other eight genes were found to be novel. NDUFB1 and NDUFA3 were reported to be associated with the OXPHOS pathway exhibited alterations in clear-cell renal-cell carcinoma [31] and lung squamous cell carcinoma [32]. NDUFB4 was associated with poor prognosis of colorectal cancer [33] and gastric cancer [34]. NDUFB8 was hypermethylated gene in glioblastoma which could be used as novel biomarkers for the prognosis [35]. The downregulation of ATP4B in plasma is a marker for gastric cancer [36]. ATP6V1G2 of the V-ATPase that were related to glioma patient prognosis [37] and was ferroptosis-related to glioma [38]. ATP12A is overexpressed in human and murine pancreatic cancer [39]. COX6B1 could be targeted by miRNA-30b to inhibit the proliferation and invasion in lung adenocarcinoma. Interestingly, CYC1 among oncogene-like genes and core biomarkers in UVM [40]. Except for CYC1, all these genes are not reported to be involved in the UVM prognosis.

Tumor microenvironment (TME) plays an important role for tumor cells to survive. The immune component of TME plays an important role in gene expression and clinical efficacy of tumor tissue [41]. UVM is different from several other tumor types, its lymphocyte infiltration indicates poor prognosis and is usually associated with metastasis [42, 43]. In this study, we found that the T cell CD8, T cells CD4 memory resting, T cell follicular helper, T cell regulatory Treg, Monocytes, Macrophages M1, Dendritic cells resting and Dendritic cells active were significantly expressed differently between the high- and low risk group. The gene signatures of CD8 + T-cell immune infiltration were reported to be used to improve prediction of the prognosis and immune response of UVM [44]. The tumor-infiltrating CD8 + T cells and macrophages were associated with better overall survival in uveal melanoma liver metastasis [45]. The GSEA analysis of the KEGG pathway based on the DEGs were enriched in oxidative phosphorylation, natural killer cell mediated cytotoxicity, leukocyte transendothelial migration, T cell receptor signaling pathway, etc., which was consistent with the immune cell infiltration analysis. The 9 OXPHOS-related prognostic risk score model was also involved in the TME immune cell infiltration.

GSEA analysis showed that various tumor related stimulation pathways were activated in prognostic high-risk score model. For example, IL-6-JAK-STAT3 is closely related to autoimmune diseases and tumors, and has become a hot target for drug research and development [46]. IL-6 activates JAK kinase after binding to its receptor. JAK kinase then catalyzes phosphorylation modification of STAT3 protein (a transcription factor) bound to the receptor. The modified STAT3 protein enters the nucleus in the form of dimer, binds to target genes, and promotes cell proliferation and differentiation [47]. MTORC1 can promote lipolysis, lipid synthesis and protein synthesis through phosphorylation, while inhibiting autophagy [48]. Besides, the pathways KRAS, TNFA- NFKB, NOTCH, and P53 were all significant pathways that participate in tumor development. It’s notable that the DEGs between the low- and high-risk group were enriched in these pathways.

The role of 9 OXPHOS related genes in cancer were reported, such as COX6B2 promoted oxidative phosphorylation, proliferation, and survival in human lung adenocarcinoma [49]. CYC1 silencing significantly reduces complex III activity and potentiates TRAIL-induced cytochrome c release and caspase-9 activation in OS cells, suggesting that CYC1 silencing acts via the mitochondria-dependent apoptotic pathway [50]. Further, we identified that CYC1 was key genes in UVM progression. CYC1 silencing significantly inhibited cell proliferation, invasion and promoted cell apoptosis in UVM cells. CYC1 acts as an oncogene role in UVM.

This study also has limitations, such as using only a public database (TCGA-UVM, GSE39717 and GSE22138) to construct and validate the prognostic model, which needs more newly diagnosed UVM patients to validate the prognostic risk score model. It is worthwhile to further investigate the role of 9 OXPHOS related gens in UVM.

## Conclusion

In conclusion, we identified 9 OXPHOS-related prognostic genes in UVM based on the lasso regression analysis to constructed prognostic risk score model of UVM. The risk model was validated in the GSE22138 dataset cohort. The risk score levels were associated with immune cell frequency and specific genomic alterations. The DEGs between the low- and high- risk group were enriched in tumor OXPHOS and immune related pathway. This OXPHOS-related prognostic risk model can help to find potential therapeutic targets for UVM patients.

### Electronic supplementary material

Below is the link to the electronic supplementary material.

Supplementary Material 1

## Data Availability

All data can be accessed by contacting the corresponding author. The datasets generated and/or analysed during the current study are available in the UCXC Xena (https://xenabrowser.net/datapages/) and GEO database (GSE22138, https://www.ncbi.nlm.nih.gov/geo/query/acc.cgi?acc=GSE22138), (GSE39717, https://www.ncbi.nlm.nih.gov/geo/query/acc.cgi?acc=GSE39717) repository.
