# Supplementary material for: Construction of oxidative phosphorylation-related prognostic risk score model in uveal melanoma
Source: BMC Ophthalmol. 2024 May 2;24:204. doi: 10.1186/s12886-024-03441-6 (PMC11067154; doi:10.1186/s12886-024-03441-6)

**Supplemental Materials**

**Figure S1**

The Univariate Cox independent prognostic analysis of 42 Oxidative phosphorylation related genes.


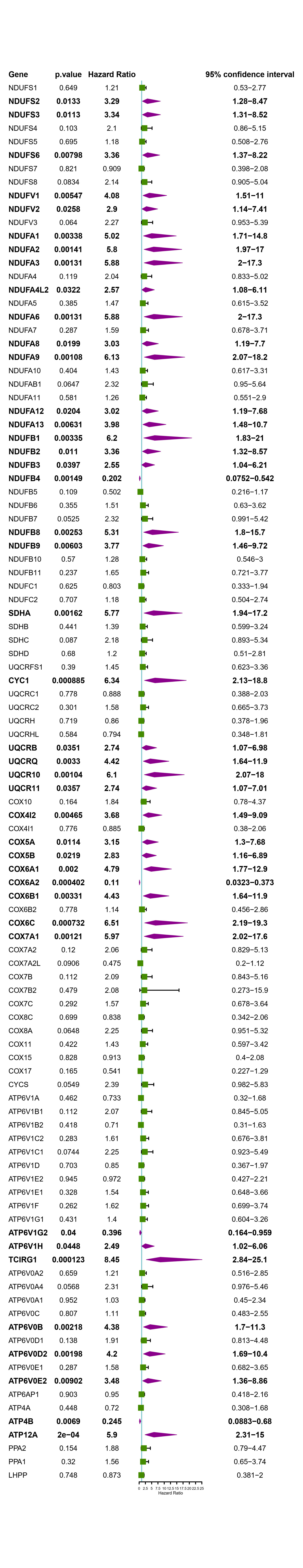


**Figure S2 The Kaplan-Meier survival curve of the 9 OXPHOS-related signature genes.**


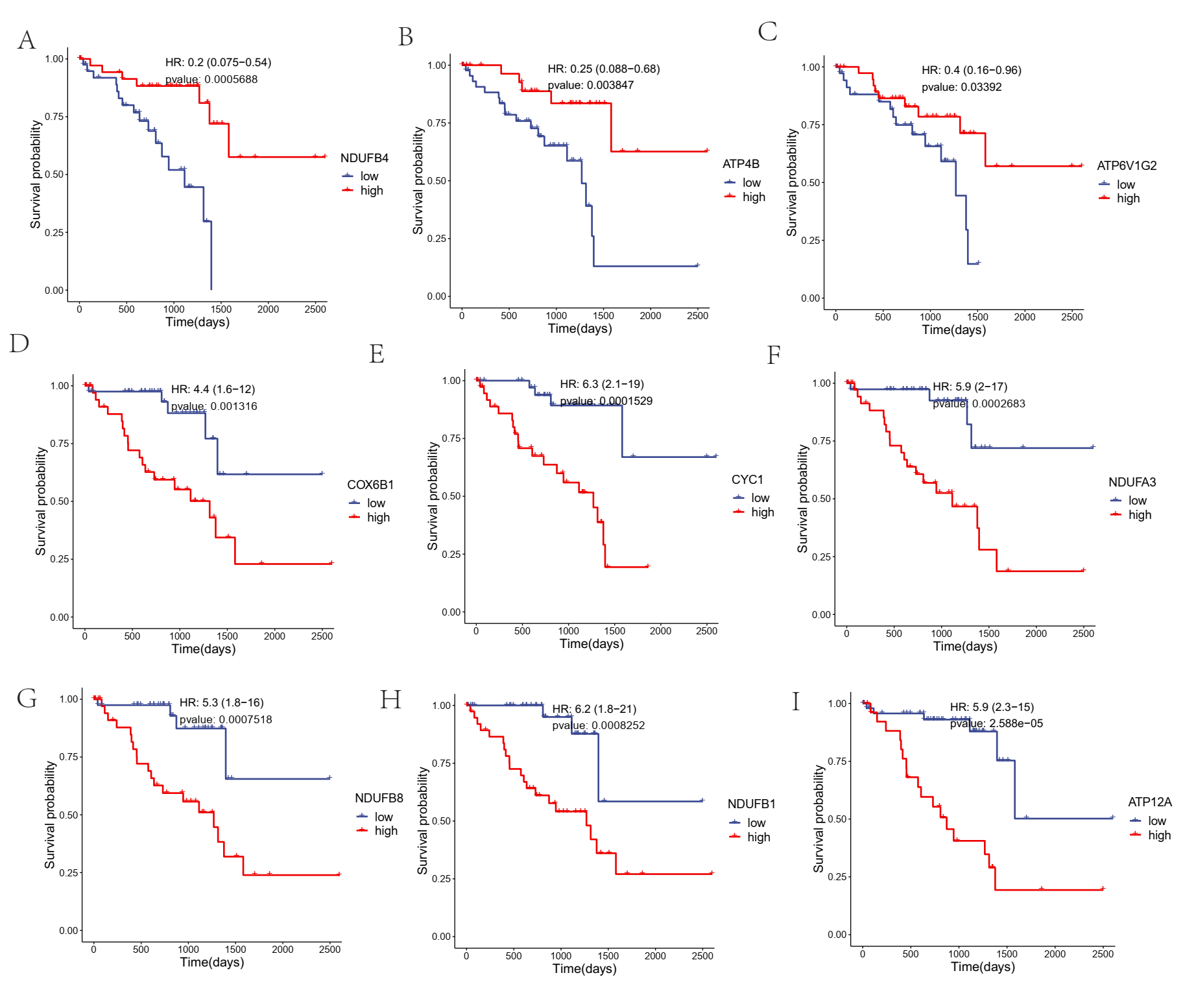


**Figure S3 Performance evaluation of prognostic risk score model in external validation sets GSE38717.**

(A) The risk score of the samples in high-risk group and low-risk group. The heatmap showing the expression level of the 9 OXPHOS-related signature genes.

(B) The Kaplan-Meier survival curve of high-risk group and low-risk group.

(C) The one to five-year Time ROC curve.


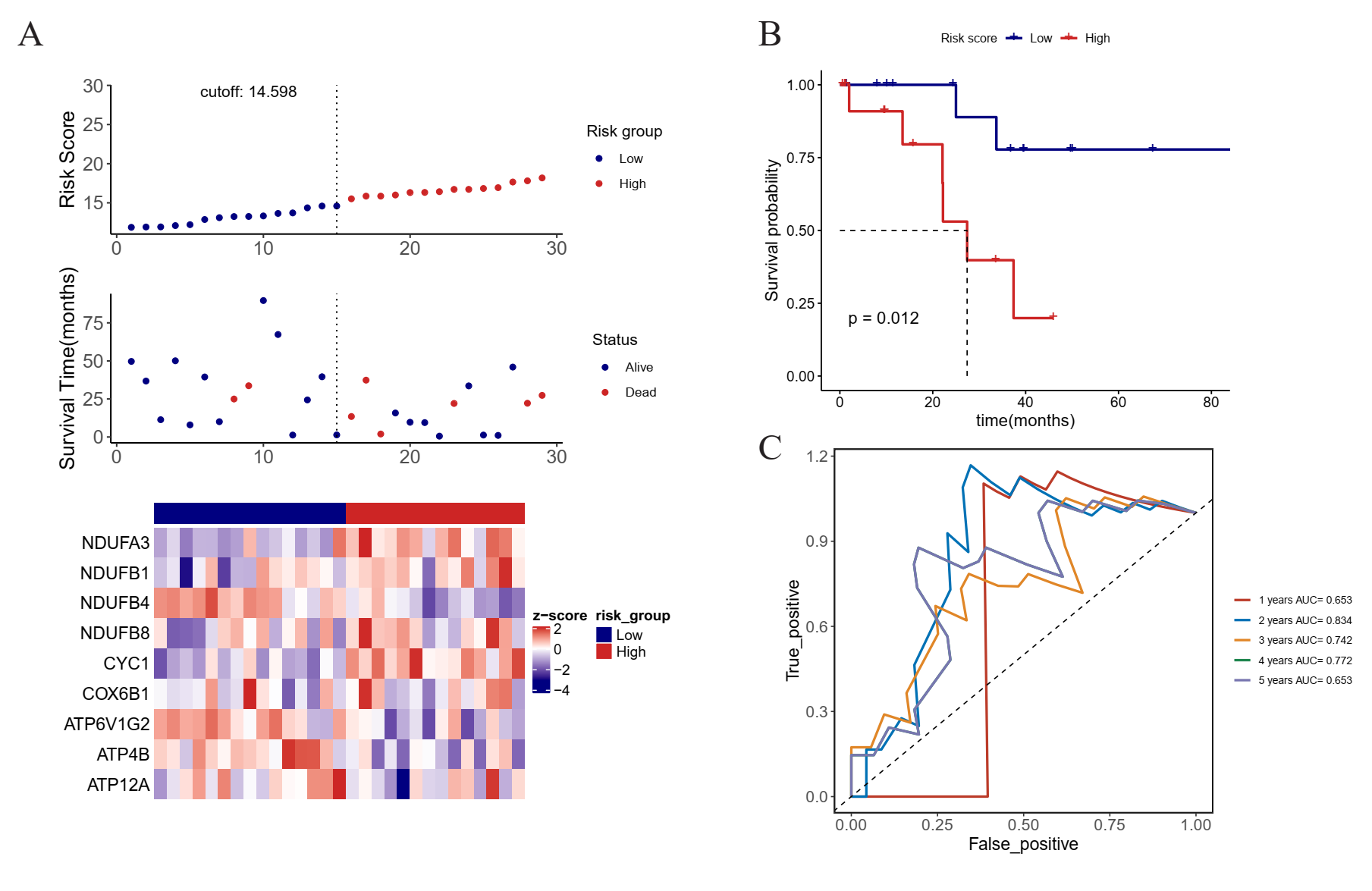

Supplement: Supplementary file 1 — Supplementary Material 1 [file 12886_2024_3441_MOESM1_ESM.docx]
